# Weighted vest interventions in older adults: a mini-review of implementation, benefits, and limitations

**DOI:** 10.3389/fpubh.2026.1811712

**Published:** 2026-03-25

**Authors:** Hongcheng Luo, Xing Zhang, Hansen Li, Mingyue Yin, Zhaoqian Li

**Affiliations:** 1 Department of Physical Education, Xichang University, Xichang, China; 2 Department of Physical Education and Sport, Faculty of Sport Sciences, University of Granada, Granada, Spain; 3 School of Physical Education, Sichuan Agricultural University, Ya’an, China; 4 School of Athletic Performance, Shanghai University of Sport, Shanghai, China; 5 School of Physical Education, Shandong University, Jinan, China

**Keywords:** bone mineral density, falls, neuromuscular function, older adults, weighted vest

## Abstract

Weighted vests (WVs) enable adjustable external loading during daily activities and exercise. Although experimental studies in older adults exist, their findings have not been comprehensively integrated. This narrative review summarizes implementation, main findings, and limitations. In October 2025, we searched Web of Science, PubMed, and EBSCOhost and conducted backward and forward citation searching. Eligible studies were peer-reviewed, English-language reports in adults aged ≥60 years. Fifteen articles were included and synthesized qualitatively. Implementation should begin with assessment and safety screening. WV use follows two modes—wear-only and task-based—and should emphasize conservative starts, gradual loading, and ongoing monitoring and adjustment. Across the limited available studies, benefits tended to be observed in task-based, progressively loaded prescriptions, including improvements in selected neuromuscular measures and, in some settings, maintenance of hip bone mineral density. Across available studies, low-load wear-only protocols generally did not demonstrate measurable improvements in musculoskeletal outcomes. Psychological gains, when present, were modest and setting-specific, and broad improvements in health-related quality of life were not observed. Although adverse events were generally mild, usability challenges and a higher number of recorded falls in one trial justify cautious implementation. Given persistent limitations in sample size, reliance on self-report, and protocol heterogeneity, adequately powered multicenter trials with objective wear-time assessment, harmonized outcomes, and extended follow-up are warranted.

## 1 Introduction

Population aging is intensifying and accelerating worldwide, particularly in high-income countries (1). According to the World Health Organization, the number of people aged 60 years and older is projected to rise from about 1.1 billion in 2023 to approximately 1.4 billion by 2030 and to around 2.1 billion by 2050, with growth expected to occur at an unprecedented pace in the coming decades (1). Age-related structural and physiological changes affect multiple systems, including the musculoskeletal, cardiovascular, respiratory, and nervous systems (2). In the musculoskeletal domain, aging is associated with declines in muscle mass and strength (3), reduced neuromuscular control (4), and deterioration of bone quantity and quality (5). These changes contribute to mobility limitations, chronic pain, impaired balance, increased fall risk, and a higher incidence of fragility fractures, thereby undermining older adults’ functional capacity and overall quality of life (6–8). They also impose substantial healthcare costs. For example, a U. S. national analysis estimated that medical spending attributable to fatal and non-fatal falls among older adults totaled about $50 billion in 2015 (9).

To mitigate the individual and societal impacts of age-related musculoskeletal decline, substantial efforts have been undertaken in both research and practice, with physical exercise widely recognized as an effective strategy (10–12). For example, a Cochrane review reported that performing progressive resistance training two to three times per week improves physical function in older adults (11). Another review found that exercise programs emphasizing balance and functional training reduce both the rate of falls and the number of fallers among community-dwelling older adults (10). Nevertheless, despite broad consensus on the benefits of exercise for musculoskeletal health, translating these benefits into meaningful real-world gains remains challenging. Exercise-related pain, fear of falling, and time or transportation barriers can undermine adherence to physical activity among older adults (13). According to recent U. S. national surveillance data, only 13.9% of adults aged 65 years and older met the federal physical activity guidelines for both aerobic and muscle-strengthening activities in 2022 (14). Therefore, further optimization of exercise prescriptions and delivery models is warranted.

Weighted vests (WVs) are wearable garments with small removable weights that allow easy, fine-tuned, task-specific loading (15–17). They can be worn over regular clothing and used during everyday functional activities (e.g., walking, stair climbing, sit-to-stand). They are designed to minimize disruption to usual movement patterns, although gait may still change depending on the load, fit, and task demands. No special equipment is required. These features make WVs simple, low-cost, and portable, and suitable for home- or community-based use. Among community-dwelling older adults, a randomized controlled trial showed that adding a vest weighing approximately 10% of body mass to a home exercise program improved strength, sit-to-stand performance, and aerobic capacity compared with exercise alone (15). In postmenopausal women, a WV jumping program was shown to help maintain hip bone mineral density (18). Regarding adherence, previous studies have reported good acceptability and completion rates, suggesting that WVs may support exercise participation in older adults (19, 20).

To date, experimental studies have made preliminary explorations of the role of WVs in older adults (15–17, 20). However, a comprehensive understanding of their findings remains limited. To address this gap, this review synthesizes the current evidence on WVs in older adults and outlines implementation strategies, main findings, and limitations.

## 2 Methods

We chose a mini-review rather than a systematic review because the evidence base in this area remains limited and heterogeneous, and our aim was to provide a broad overview of the implementation, main findings, and limitations of WVs in older adults rather than to synthesize a single prespecified outcome. In October 2025, we performed a targeted search of Web of Science, PubMed, and EBSCOhost using combinations of the following terms and wildcards: weight* vest, load* vest, wearable resistance, aged, older, elderly, older adult*. Database-specific search strategies and database-specific yields are provided in Supplementary Table S1. To enhance completeness, we performed backward citation searching (reference lists of included studies and relevant reviews) and forward citation searching in Google Scholar (“Cited by”). Eligible records were peer-reviewed, English-language studies examining weighted vests (WVs) in older adults (age ≥60 years). We excluded protocols, conference abstracts without full data, editorials, and non-human studies. For this narrative mini-review, two reviewers independently screened titles/abstracts and full texts, resolving disagreements by consensus. Fifteen studies met the eligibility criteria and were included in the synthesis (7, 15–28); their characteristics are summarized in Supplementary Table S2.

## 3 Definition and rationale

**Figure 1:**
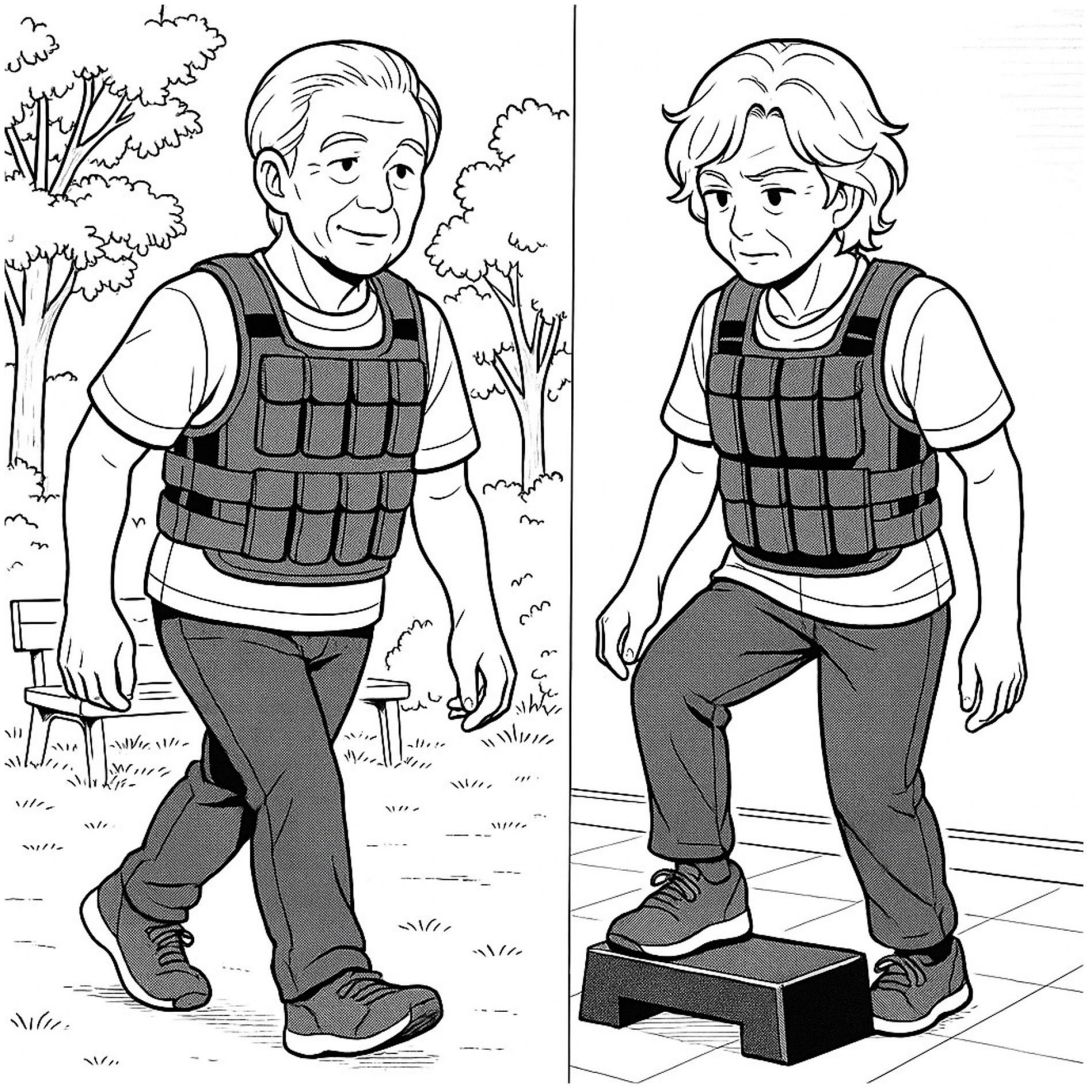
Illustration of weighted-vest training in older adults: an adjustable, pocket-loaded weighted vest used during walking (left) and step-up training (right) in older adults.

A WV is a wearable garment—typically a nylon vest—with multiple pockets that hold removable small loads (Figure 1). It enables fine-tuned external loading during activity while aiming to preserve the basic movement pattern. In geriatric trials, WVs have been prescribed as either (i) absolute loads (26) or (ii) a percentage of body mass (e.g., 3–10%) (15). Early studies used pocketed WVs worn for set durations (e.g., 2 h per day, 4 days per week) (25). Later protocols paired the WV with functional tasks—walking, stair or step work, sit-to-stand, squats, or impact/jumping—to increase task-specific mechanical loading (16, 18).

## 4 Implementation

### 4.1 Assessment and safety screening

Implementation of WVs in older adults should begin with assessment and safety screening. Candidates should have sufficient cognitive capacity and physical ability to manage the vest (don and doff) and to ambulate safely (15). Planned WV-augmented tasks should align with the individual’s functional status (e.g., sit-to-stand, level walking, step-ups or stairs) (15). Before initiation, screen for conditions that require caution or deferral, including recent fracture or an acute musculoskeletal flare, uncontrolled cardiopulmonary disease, severe balance or gait disorders, and target-joint pain likely to limit participation (26). A supervised familiarization is recommended (including don/doff practice), starting on flat surfaces and avoiding complex or higher-risk terrain (e.g., stairs, uneven ground), while ensuring compatibility with any assistive devices.

### 4.2 Exercise prescription

Use of WVs in older adults can follow two modes: wear-only and task-based. In the wear-only mode, the vest is incorporated into daily life, and older adults wear it in prescribed time blocks while carrying out usual activities without structured exercise. For example, some protocols prescribe 2 h/day on 4 days/week at 3–5% of body mass (25). In the task-based model, older adults integrate WVs into structured exercise sessions. For example, they may train 2–4 times per week in sessions lasting about 20–40 min (16, 21, 22). A typical session includes a warm-up, a task block (e.g., walking, step-ups/stair climbing, sit-to-stand, or squats), and a cool-down (16, 21, 22).

Load planning in WV programs generally follows a progressive loading principle. Begin at 1–5% of body mass (frail or first-time users toward 1–3%; average function 3–5%) (25). If technique and gait remain stable, increase the vest load weekly in small steps to achieve progressive overload (20). Loads should be task specific. In structured sessions, 5–10% of body mass typically provides sufficient osteogenic and myomechanical stimulus (e.g., vest-loaded squats) (16, 18, 25). By contrast, a wear-only, home-based protocol using 5% of body mass for 2 h/day on 4 days/week did not observe improvements in strength, function, or bone turnover, which may indicate that 5% body mass without structured activity is not a sufficient stimulus in some older-adult samples (20). Therefore, scale the load to task difficulty: use the week’s highest tolerated load for level-ground walking, reduce for step-ups or stairs, and reduce further or use body weight only for balance drills.

### 4.3 Monitoring and adjustment

**Figure 2:**
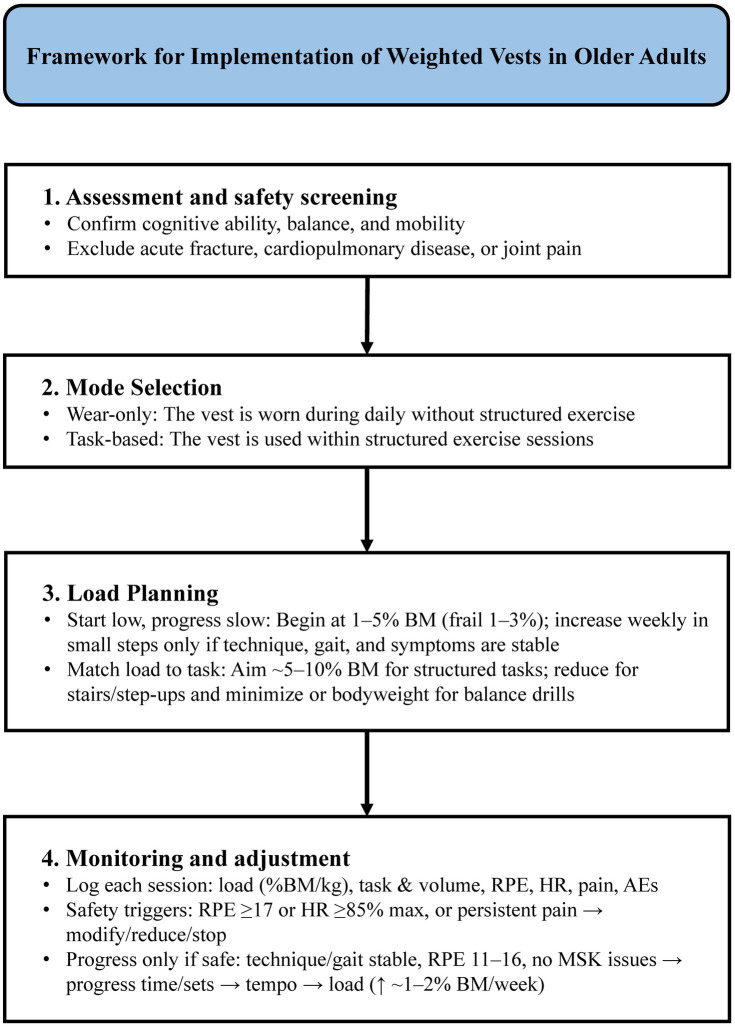
Framework for implementation of weighted vests in older adults. Stepwise framework covering screening, mode selection (wear-only vs. task-based), load planning, and monitoring/adjustment (RPE/HR, symptoms, adverse events).

Monitoring and adjustment are essential for setting exercise load and ensuring safety during WV training. At each session, record the vest load (kg or % body mass), the task and volume (time/sets/tempo), RPE, heart rate, pain, and any adverse events (15, 21, 22, 28). These indices can guide day-to-day load adjustment and session management. For example, some protocols reduce or terminate sessions when exertion exceeds the intended range (e.g., RPE ≥ 17) or when heart rate is high relative to age-predicted maximum values (e.g., ≥ 85%) (21, 22, 27). Apply clear stop rules (e.g., new/worsening pain, dizziness, marked instability/near-falls) and reassess the dose or setting if triggered. If persistent local pain or discomfort occurs, modify the exercise (e.g., technique or external support), reduce the load, or omit the exercise (21, 22, 27). Progress only when technique is stable, gait is steady, RPE is within the target range, and there are no persistent musculoskeletal complaints (21, 22, 27). To minimize adverse responses, apply a “time/sets → tempo → load” progression. Consider small weekly load increments, such as increasing vest mass by ~1–2% of body mass when tolerance criteria are met. The implementation process for WVs is presented in Figure 2.

## 5 Main findings

### 5.1 Bone health

The effects of WVs on bone health in older adults are moderated by the training prescription. Adequate stimulus—via load, volume, and exercise selection—is important. For example, Snow et al. (18) added WVs to functional exercises such as stepping, squats, chair stands, forward and lateral lunges, toe raises, and jumps. Over a 5-year intervention (32 weeks/year, 3 sessions/week) with an average vest load of 11.3 lb., the WV group showed greater improvement and maintenance of hip bone mineral density than a control group that continued usual lifestyle and weight-bearing activities (femoral neck: +1.54% ± 2.37 vs. − 4.43% ± 0.93; trochanter: −0.24% ± 1.02 vs. − 3.43% ± 1.09; total hip: −0.82% ± 1.04 vs. − 3.80% ± 1.03). A shorter-term study likewise found significant gains in femoral-neck bone mineral density in community-dwelling older women (69.2 ± 3.5 years) (24). Over 32 weeks, participants completed 3 sessions/week of functional plus resistance training, with vest load progressively increased to 10% of body mass. However, when WV stimulus is lower, bone-related benefits may be attenuated. In a 20-week, once-weekly program that combined functional exercise with daily wear, relatively light loads were used (max 3.6 kg) (26). Under these conditions, only a trend toward greater dual-energy X-ray absorptiometry–measured bone mineral density was observed in the WV group versus a health-education control (+1% vs. − 0.6%, *p* = 0.12). Greendale et al. (25) used 3% or 5% of body mass for daily wear only (4 days/week, 2 h/day) and found no improvement in bone turnover markers; the authors suggested that this dose may have been insufficient to provide an osteogenic stimulus. In practice, WV prescriptions should therefore be tailored to participant characteristics and provide adequate stimulus to obtain bone health benefits.

### 5.2 Neuromuscular function

Regarding neuromuscular function, Greendale et al. (25) examined a wear-only model in older adults. Participants wore WVs at 3 or 5% of body mass for 2 h/day on 4 days/week over 27 weeks. The intervention did not improve neuromuscular outcomes, including isometric and isokinetic knee extensor/flexor strength, muscular endurance, or functional performance measures (walking, chair rises, stair climbing, balance). These null findings may be attributable to the combination of low vest loads and the wear-only model. However, given the small number of trials and variable protocols, certainty is limited. Moreover, higher-load wear-only models have not been adequately tested in older adults, so their effects on neuromuscular outcomes remain unclear. By contrast, findings from task-based models are more favorable. Mierzwicki (15) prescribed 12 weeks of home-based functional training, 3 sessions/week, with participants wearing WVs loaded to 10% of body mass during all training and walking. The intervention produced significant pre- to post-intervention improvements across all 12 assessed neuromuscular outcomes, including the five-times sit-to-stand test, 30-s chair-stand test, 2-min step test, 6-min walk test, single-leg heel raises, and isometric hip-muscle strength. Notably, another study with a simpler program and shorter duration also observed positive effects of WVs on neuromuscular function. Specifically, Mair et al. (23) implemented a 6-week step-training protocol, 3 sessions/week, with progressive vest loading up to 10% of body mass. This approach improved lower-limb power and stair-climb performance in older adults, whereas isometric strength and general functional measures (chair rises, habitual-speed walking) showed no significant change. This pattern likely reflects the principle of specificity—that is, specific adaptations to the imposed demands in exercise (29–31). Because the intervention required step-ups performed as fast as possible, gains were more likely to transfer to lower-limb power and stair-climb performance than to isometric strength or general functional measures. Therefore, when designing WV interventions for older adults, exercise specificity should be carefully considered.

Several studies suggest that combining weighted vests (WVs) with adjunct interventions may enhance outcomes. In older adults, squats on a whole-body vibration platform (30 Hz, 2 mm) while wearing a vest loaded to 10% of body mass improved isometric strength, quadriceps thickness/muscle mass, balance, and functional mobility, with balance/mobility gains exceeding those from WV or vibration alone (16). Nutritional co-interventions may also add value: an 8-week program pairing progressive WV loading (5–10% body mass) with chicken-protein supplementation improved multiple functional outcomes and produced greater handgrip gains than WV alone, suggesting a potential benefit for upper-limb strength proxies (7). One study further reported that during 22 weeks of energy restriction (~1,100–1,300 kcal/day), vest wear (~6.7 h/day) with load titrated to weight loss (up to 15% of baseline body mass) helped preserve bilateral leg power compared with energy restriction without a vest (19). Overall, early evidence indicates possible additive effects—particularly for balance/mobility and handgrip strength—but small samples and heterogeneous protocols limit generalizability and warrant further trials.

### 5.3 Psychological outcomes

Weighted vest interventions may also influence psychological outcomes, but evidence remains limited and inconsistent. Greendale et al. (26) implemented a 20-week program combining once-weekly functional exercise with daily vest wear using relatively light loads (max 3.6 kg). Compared with a health-education control, the intervention reduced Multidimensional Health Locus of Control (MHLC) “Powerful Others” and “Chance” scores (*p* = 0.05 and 0.04), suggesting less external attribution; however, effects were borderline and the MHLC “Internal” subscale did not increase. In a separate trial, Greendale et al. (25) tested a wear-only model (3% or 5% of body mass, 4 days/week, 2 h/day) and found MHLC changes only on “Powerful Others,” with the 5% group showing a more external orientation at follow-up. For health-related quality of life, Hakestad et al. (28) combined WVs with functional training and assessed the 36-Item Short Form Health Survey (SF-36) outcomes, but the intervention did not outperform a non-exercise control at 6 months or at 12-month follow-up. A similar null finding was reported with the wear-only protocol above using the SF-36 (25). Finally, Jessup et al. (24) assessed osteoporosis-related self-efficacy after 32 weeks of resistance training plus WV functional training and observed no advantage over usual lifestyle. Overall, psychological effects—when observed—appear modest and context-dependent, while broad improvements in quality of life have not been demonstrated; larger trials with sufficient mechanical stimulus and harmonized measures are needed.

### 5.4 Contextual modifiers

Based on the current evidence, the effects of WVs are likely context-dependent. Postmenopausal/osteopenic women may require higher, task-based mechanical stimuli to meaningfully target hip bone mineral density, whereas low-load wear-only exposure may be insufficient (18, 24–26, 28). Mobility limitation and frailty may increase the need for conservative loading, clear stop rules, and supervision (21, 22, 27). During dietary weight loss (negative energy balance), WV loading titrated to weight change may help preserve leg power but demands close monitoring for discomfort and adherence barriers (17, 19, 20).

## 6 Limitations of weighted-vest interventions

### 6.1 Insufficient stimulus in wear-only models

Weighted vest effects appear highly dependent on intervention mode and dose. Wear-only protocols—particularly with low loads—often provide an inadequate mechanical stimulus, which may contribute to the largely null findings for muscle function and bone outcomes. For instance, Greendale et al. (25) prescribed vests at 3% or 5% of body mass during daily activities (4 days/week, 2 h/day) and reported no improvements in neuromuscular function or bone health, and the authors attributed these findings to an insufficient stimulus. Similarly, a largely wear-based program with only once-weekly functional training and a low load cap (max 3.6 kg) also failed to improve neuromuscular or bone outcomes (26). Overall, based on the limited available trials, simply wearing low-to-moderate loads during daily activities may not provide a sufficient training stimulus for measurable musculoskeletal adaptations in older adults.

### 6.2 Adverse events during weighted-vest use

Although serious injuries are rarely reported, several adverse events and usability issues deserve attention. In Mierzwicki (15), older adults completed home-based functional training plus 30 min walking (3×/week) while wearing a vest loaded to 10% of body mass; ~50% reported difficulty donning/doffing the vest, which the authors linked to the lack of a gradual acclimation/load-progression phase. Thus, studies that require independent vest use should incorporate a ramp-up period and select user-friendly vest designs, while monitoring and reporting minor discomfort. In a 22-week diet-induced weight-loss trial (daily wear; weekly increases up to 15% of baseline body mass), five participants reported vest-related back pain/soreness (19). Management was individualized: two stopped near weeks 19–20, two paused for 2–3 weeks then resumed, and one reduced the load and stopped progression after week 11, with symptoms resolving. This supports a pragmatic approach—temporarily stop, reduce load, and resume only after symptoms improve. Finally, Greendale et al. (25) reported one severe contact dermatitis case after wearing the vest in water, leading to discontinuation. Participants should therefore be briefed on vest materials and practical precautions (e.g., avoid water exposure if contraindicated, use a base layer, and clean/dry the vest after sweating) to reduce dermatological risk.

### 6.3 Interference with daily life

Wear-only WV protocols often require prolonged daily wear, which may disrupt everyday routines. For instance, Beavers et al. (17) reported an average wear time of 7.1 h/day in older adults. Extended wear may be uncomfortable or socially inconvenient (e.g., visible bulging under clothing at gatherings, discomfort from chair backs or seat belts when seated, and the need for frequent adjustments or explanations that increase self-consciousness). Consistent with this, Normandin et al. (19) found that 7 of 17 participants (41.2%) rated vest wear as hindering (1–2 on a 5-point scale). These findings suggest that improved vest design and/or alternative loading strategies may be needed to reduce day-to-day burden in wear-only approaches.

### 6.4 Risk of falls

In theory, WVs may increase balance or gait demands and, in unsupervised home settings, could plausibly increase the likelihood of mishaps in some older adults (32, 33). In Beavers et al. (17), falls were recorded as adverse events (14 in diet + WV vs. 10 in diet-only and 7 in diet + progressive resistance training). Because the trial was not powered for falls and no between-group hypothesis testing was reported, these data should be interpreted as a safety signal rather than evidence of a causal effect. This underscores the importance of careful prescription and monitoring.

### 6.5 Ethics and equity considerations

In addition, an ethics and equity lens is important for real-world home/community implementation. Affordability and access to appropriate vest designs/sizes, along with usability demands (e.g., donning/doffing and load adjustment), may disproportionately burden older adults with dexterity limitations, pain, or limited caregiver support, thereby affecting uptake, adherence, and safety. These equity-related constraints should be considered when translating WV guidance into practice (34).

## 7 Limitations of this review and the evidence base

Several limitations should be acknowledged when interpreting this study’s findings. This review deliberately focuses on older adults, so its inferences should not be generalized to other populations (e.g., younger adults or athletes). Because the evidence base remains small and heterogeneous—with diverse prescriptions, populations, and outcomes—we intentionally conducted a narrative review to provide a broad overview of implementation, main findings, and limitations rather than conducting a meta-analysis of a single prespecified outcome. Accordingly, our conclusions are based on qualitative synthesis and do not include pooled effect estimates.

## 8 Conclusion

Effective WV use in older adults should start with safety screening, prioritize task-based training over wear-only protocols, and apply conservative initial loads (~1–5% body mass), progressing only when gait/technique are stable while monitoring RPE, heart rate, pain, and adverse events. When integrated into structured functional or resistance exercise, WVs may improve selected neuromuscular outcomes and, in some settings, help preserve bone mineral density. In contrast, across available studies, low-load wear-only protocols generally did not demonstrate measurable improvements in muscle- or bone-related outcomes. One plausible explanation is that the mechanical stimulus may be insufficient; however, higher-load wear-only protocols have not been adequately tested in older adults. Psychological effects appear modest and context-dependent, and improvements in overall health-related quality of life have not been demonstrated. Overall safety appears acceptable, but usability barriers, localized discomfort, and a higher fall count in one trial reinforce the need for gradual progression and close monitoring. Future work should prioritize adequately powered multicenter trials with objective adherence/wear-time measures, standardized outcomes, longer follow-up, and focused evaluation in high-risk populations.
