# Supplementary material for: Weighted vest interventions in older adults: a mini-review of implementation, benefits, and limitations
Source: Front Public Health. 2026 Mar 25;14:1811712. doi: 10.3389/fpubh.2026.1811712 (PMC13056602; doi:10.3389/fpubh.2026.1811712)
Supplement: Supplementary file 1 [file Data_Sheet_1.docx]

Supplementary Material

# Table S1. Database-specific search strategies

| Database | Date | Key work | Number of articles |
| --- | --- | --- | --- |
| Web of Science | 2025/10/10 | #1 TS=(weight* vest) OR TS=(load* vest) OR TS=(wearable resistance)  #2 TS=(aged) OR TS=(older) OR TS=(elderly) OR TS=(older adult)  #3 #1 and #2 | 398 |
| PubMed | 2025/10/10 | #1 "weight* vest"[Title/Abstract] OR "load* vest"[Title/Abstract] OR "wearable resistance"[Title/Abstract]  #2 "aged"[Title/Abstract] OR "older"[Title/Abstract] OR "elderly"[Title/Abstract] OR "older adult"[Title/Abstract]  #3 #1 and #2 | 39 |
| EBSCOhost | 2025/10/10 | #1 AB (weight* vest) OR AB (load* vest) OR AB (wearable resistance)  #2 AB (aged) OR AB (older) OR AB (elderly) OR AB (older adult)  #3 #1 and #2 | 152 |

# Table S2. Characteristics of included studies

| Author | Study Design | Sample Size & Gender | Age & Health Status | Groups (Intervention vs Control) | Intervention Details | Load & Progression | Frequency & Duration | Intervention Period | Primary Outcomes | Secondary Outcomes | Main Findings | Compliance | Adverse Events |
| --- | --- | --- | --- | --- | --- | --- | --- | --- | --- | --- | --- | --- | --- |
| Jonathan Bean, Seth Herman (1) | RCT (evaluator-blinded; pilot) | 45 randomized; 40 analyzed (20/20); 34F/11M | ≥65 y; community-dwelling; mobility-limited | Vest stair-climbing vs walking | Stair-climbing training vs walking program | Vest load ↑ by +2% BW when criteria met; ~90.9% max stair-climb power | Vest: 3×/wk, ~10 min/session; Control walking progressed 15→45 min | 12 wk | Leg power; knee extensor power/strength; stair-climb power | 6MWT; functional/mobility tests; RPE/physiology | Vest stair-climbing ↑ leg power (~+17%) and stair power (~+12%); walking better on 6MWT | ~91% vs ~87% | None reported |
| Bean, Herman (2) | Single-blind RCT (pilot) | 21 (all female); 10 vs 11 | ≥70 y; community-dwelling; mobility-limited | High-velocity, task-specific vest training vs low-intensity seated/chair exercise | Supervised multi-exercise program (chair stand/step-ups + upper-body) | Start 0 load; ± load by RPE (↑ +2% BW if RPE≤16; ↓ −1% if not) | 3×/wk, ~30 min/session | 12 wk | Leg press power (multi-loads); 1RM; mobility/balance tests | Baseline health/mood scales | Vest training ↑ leg power and improved chair-stand/gait/balance vs control; both groups improved some mobility indices | ~90% vs ~88% | No serious AEs; minor shoulder pain in control; 1 pre-training dropout (transport) |
| Mair, De Vito and Boreham (3) | Quasi-experimental waitlist cross-over (within-subject) | 11 (all female) | 65–74 y; community-dwelling; low habitual exercise; healthy | 6-wk usual-life control → 6-wk home weighted step training | Home-based rapid step-up protocol (20 cm) | Load progressed 0% → 5% → 7.5% → 10% BW | 3 days/wk, 3 sessions/day (9 sessions/wk) | 6 wk intervention (+6 wk control phase) | Peak leg power; stair performance | Knee extensor strength; gait/chair-stand; HR/BP | ↑ peak power (~+10%) and improved stair performance vs control period | ~97% | None reported |
| Mierzwicki (4) | RCT (pilot) | 19 randomized; 17 completed (8 vs 9); 9M/10F | Mean 68.1±5.8 y; community-dwelling; medically cleared | Home exercise + walking with vest vs same without vest | Home functional lower-limb exercise + 30-min walk | Fixed 10% BW throughout (no progression) | 3×/wk; exercise session + 30-min walk | 12 wk | Hip strength; sit-to-stand/chair-stand; 2-min step; 6MWD | Heel-raise; brief acceptability interview | Greater improvements in chair-stand, 2-min step, 6MWD, and selected hip strength vs control; some outcomes improved in both groups | ~93% vs ~94.8% | No injuries reported; ~50% reported don/doff difficulty (needed assistance) |
| Snow, Shaw (5) | Non-randomized long-term controlled follow-up | 18 (all postmenopausal women); 9 vs 9 | Postmenopausal; groups comparable at baseline; non-smokers | Vest + jumping/resistance training vs active lifestyle (no vest/jumping) | Long-term jumping + lower-limb training program | Mean vest ~11.3 lb (~5.1 kg); no fixed % progression reported | ~3×/wk; ~32 wk/year (session duration NR) | 5 y | Hip BMD (DXA) | Dietary Ca/Vit D monitoring | Intervention maintained hip BMD vs declines in control (significant between-group differences) | ~83.6% | No new injuries reported |
| Jessup, Horne (6) | RCT (assessor-blinded; pilot) | 20 randomized; 18 completed (9 vs 9); all female | Postmenopausal; mean age 69.2±3.5 y; sedentary; Ca/Vit D supplementation | Multicomponent exercise + weighted vest vs usual activity (no exercise) | Supervised resistance/functional + balance program including weighted walking/stairs | 0 load first 2 wks; then +1 kg/week to ~10% BW (over 4–6 wks) | 3×/wk; 60–90 min/session | 32 wks | Hip/lumbar BMD; postural sway; total strength | Grip strength; self-efficacy; body mass | Greater ↑ femoral-neck BMD, ↓ sway, and ↓ body mass vs control; no group difference for lumbar BMD or total strength | ~90% completion (18/20) | None reported (dropouts unrelated: health issue; relocation) |
| Greendale, Salem (7) | RCT (non-blinded; home-based wear-only) | 62; ~74% female | Mean age ~74 y; community-dwelling; independently ambulatory | No vest vs 3% BW vest vs 5% BW vest | Wear vest during daily activities (no structured exercise) + logs/follow-up | Fixed 3% or 5% BW; 3-wk ramp-up then prescribed wear | 4 d/wk; 2 h/day | 27 wks | Isokinetic knee extensor strength | Physical performance; bone turnover markers; HRQoL; falls efficacy; MHLC | No meaningful between-group improvements in strength, function, bone markers, or HRQoL; only a small MHLC domain difference (inconsistent direction) | Follow-up completion ~80–91%; “ideal adherence” ~55–58% | 3 discontinuations (loss of interest, back pain, contact dermatitis in water); otherwise no serious AEs |
| Kelleher, Beavers (8) | RCT (pilot) | 37 randomized; 33 completed; ~78% female | 65–79 y; obese; sedentary | Diet-induced weight loss + daily vest wear vs diet-only | 22-wk energy restriction with counseling; vest worn during most active time | Load titrated with weight loss; up to 15% BW; mean ~7.1% BW | Target 10 h/day; actual ~6.7 h/day | 22 wks | Hip/lumbar areal BMD; bone biomarkers | Body mass change; adherence/process measures | Similar weight loss between groups; no significant between-group differences in primary outcomes, but trends favored vest for less hip BMD loss and higher bone formation marker | High diet adherence; vest wear ~6.7 h/day; target-day proportion ~67% | No serious intervention-related AEs reported |
| Beavers, Lynch (9) | Single-blind RCT (parallel groups) | 150 randomized; 133 completed; ~75% female | 60–85 y; obese; community-dwelling | Diet-only vs diet+vest vs diet+progressive RT | All: ~10% weight-loss program; vest worn during active time; RT supervised | Vest load adjusted weekly to replace lost mass (≤10% baseline BW) | Vest target 8 h/day; RT 3×/wk; 12 mo program | Hip trabecular vBMD (QCT); hip areal BMD (DXA) | Other skeletal outcomes; bone biomarkers; body comp; strength; MVPA | All groups lost similar weight; hip BMD declined in all groups; diet+vest not different from diet-only; neither vest nor RT prevented hip bone loss (RT improved some secondary outcomes) | Vest wear ~7.1 h/day; RT attendance ~71% | 193 AEs (mostly “other”/musculoskeletal); musculoskeletal AEs higher in vest/RT; 6 serious AEs unrelated | |
| Srisaphonphusitti, Manimmanakorn (10) | RCT (assessor-blinded) | 60 randomized; 51 completed; 11M/40F | 60–80 y; healthy; mean age 65.6±3.8 y | WBV vs vest-squat vs WBV+vest-squat | Squat-based sessions; WBV platform used in relevant arms | Vest: week 1 ~0.5 kg; week 2 5% BW; weeks 3–8 10% BW | 3×/wk; 10×1-min sets/session | 8 wks | Max isometric strength | Muscle mass/thickness; balance; TUG; HR/BP | Strength ↑ in all groups; balance and TUG improvements most pronounced in WBV+vest; muscle mass ↑ similarly | Attendance ~90% | No training-related AEs reported |
| Nithisup, Manimmanakorn (11) | RCT (3-arm parallel; allocation concealed) | 52 completed (all female): 17/19/16 | 60–79 y; community-dwelling; baseline characteristics comparable | Exercise (no vest) vs exercise+vest vs exercise+vest+protein | Standardized mixed exercise session (warm-up + brisk walk + resistance circuit) | Vest: 5% BW (wks 1–2) → 10% BW (wks 3–8); protein ~3 g/day | 3×/wk; ~30 min/session | 8 wks | Lean mass; BMC/BMD (DXA; incl. T-score) | Fat mass; glucose/lipids; grip; function (6MWT, TUG, STS) | Vest+protein improved lean mass and thoracic spine BMC vs control/vest-only; vest-only showed largest 6MWT gain; both vest groups had higher BMD T-scores vs control | ≥90% session completion; protein taken daily per protocol | No training-related AEs reported |
| Greendale, Hirsch and Hahn (12) | RCT (community senior center) | 36 randomized (19 vs 17); follow-up ~83% | Mean age ~70–73 y; community-independent; key exclusions for major comorbidity | Low-intensity class + home vest wear vs health education (no vest) | Weekly low-intensity class + home wear during daily activities | 0.45 kg start; ↑ up to 0.9 kg/week to max 3.6 kg (mean ~1.1 kg) | Class 1×/wk; home wear ~3–4 d/wk, ~1 h/day (tolerated) | 20 wks | Lumbar spine BMD; SF-20 domains | Health locus of control; morale; chair-stand | Vest group reported less pain and better physical function; lumbar BMD showed a small favorable change (between-group NS); greater wear time correlated with BMD change | Class attendance ~84–88%; diaries ~84% | Mild AEs: knee flare (withdrawal) and upper-back pain (resolved with load reduction); no serious AEs |
| Hakestad, Torstveit (13) | Single-blind RCT; orthopedic follow-up; 1-y follow-up | 80 women randomized (42 vs 38); 1-y follow-up 31 vs 33 | Postmenopausal; low BMD (T < −1.5); healed distal radius fracture | OsteoACTIVE (exercise+vest + education) vs education only | Multicomponent supervised + home program (strength/balance/core/coordination) | Vest used; load progression not reported | 3×/wk; 60 min/session (2 supervised + 1 home) | 6 mo; follow-up to 1 y | Quadriceps isokinetic strength; hip/lumbar BMD | Balance; 6MWT; PA; SF-36 | No significant between-group differences for strength, BMD, function, PA, or HRQoL at 6 mo or 1 y | Attendance ~87%; 72% met ≥80% attendance | No intervention-related AEs reported |
| Bean, Kiely (14) | Single-blind RCT; supervised training comparison | 138 randomized; 117 completed; ~69% female | ≥65 y; mobility-limited (SPPB 4–10) | High-velocity task-specific vest training vs NIA strength training (free weights) | Supervised group sessions with standardized monitoring | Vest: +2% BW increments; control weights +1–2 lb | 3×/wk; 45–60 min/session | 16 wks | Limb power; SPPB total score | 1RM; estimated limb velocity; self-reported function | Vest training produced greater gains in limb power; both groups improved strength and SPPB similarly; functional self-reports improved in both | Attendance ~81% vs ~79% | No serious AE differences; 1 non-injury fall/group; training modifications for MSK discomfort; a few dropouts (flu/myalgia) |
| Normandin, Yow (15) | RCT (pilot; single-center) | 37 randomized; 33 completed; ~78% female | 65–79 y; obese; sedentary | Diet-induced weight loss + daily vest wear vs diet-only | Energy restriction program; no structured exercise | Vest load titrated with weight loss (≤15% baseline BW; mean ~7.1% BW) | Target 10 h/day; actual ~6.7 h/day | 22 wks | Body composition (DXA); function; limb power | Waist/hip; satisfaction/feasibility | Similar weight loss; vest group preserved lower-limb power vs decline in control; other functional outcomes largely similar | Wear time ~6.7 h/day; retention ~89–90% | Back pain/soreness in some participants; some discontinuations/pauses; no serious AEs reported |

Note: RCT - Randomized Controlled Trial, BW - Body Weight, 6MWT - 6-Minute Walk Test, 1RM - One Repetition Maximum, SPPB - Short Physical Performance Battery, BMD - Bone Mineral Density, DXA - Dual-Energy X-ray Absorptiometry, PA - Physical Activity, HRQoL - Health-Related Quality of Life, TUG - Timed Up and Go, MHLC - Multidimensional Health Locus of Control, BMC - Bone Mineral Content.

References

1. Jonathan Bean M, Seth Herman B, Mph DKK, Damien Callahan B, Kelly Mizer B, Md WRF, Fielding RA. Weighted Stair Climbing in Mobility‐Limited Older People: A Pilot Study. *Journal of the American Geriatrics Society* (2002) 50(4):663-70.

2. Bean JF, Herman S, Kiely DK, Frey IC, Leveille SG, Fielding RA, Frontera WR. Increased Velocity Exercise Specific to Task (Invest) Training: A Pilot Study Exploring Effects on Leg Power, Balance, and Mobility in Community‐Dwelling Older Women. *Journal of the American Geriatrics Society* (2004) 52(5):799-804.

3. Mair JL, De Vito G, Boreham CA. Low Volume, Home-Based Weighted Step Exercise Training Can Improve Lower Limb Muscle Power and Functional Ability in Community-Dwelling Older Women. *Journal of clinical medicine* (2019) 8(1):41.

4. Mierzwicki JT. Weighted Vest Training in Community-Dwelling Older Adults: A Randomized, Controlled Pilot Study. *Physical Activity and Health* (2019) 3(1).

5. Snow CM, Shaw JM, Winters KM, Witzke KA. Long-Term Exercise Using Weighted Vests Prevents Hip Bone Loss in Postmenopausal Women. *The Journals of Gerontology Series A: Biological Sciences and Medical Sciences* (2000) 55(9):M489-M91.

6. Jessup JV, Horne C, Vishen R, Wheeler D. Effects of Exercise on Bone Density, Balance, and Self-Efficacy in Older Women. *Biological Research for nursing* (2003) 4(3):171-80.

7. Greendale GA, Salem GJ, Young JT, Damesyn M, Marion M, Wang MY, Reuben DB. A Randomized Trial of Weighted Vest Use in Ambulatory Older Adults: Strength, Performance, and Quality of Life Outcomes. *Journal of the American Geriatrics Society* (2000) 48(3):305-11.

8. Kelleher JL, Beavers DP, Henderson RM, Yow D, Crotts C, Kiel J, et al. Weighted Vest Use During Dietary Weight Loss on Bone Health in Older Adults with Obesity. *Journal of osteoporosis and physical activity* (2017) 5(4):210.

9. Beavers KM, Lynch SD, Fanning J, Howard M, Lawrence E, Lenchik L, et al. Weighted Vest Use or Resistance Exercise to Offset Weight Loss–Associated Bone Loss in Older Adults: A Randomized Clinical Trial. *JAMA Network Open* (2025) 8(6):e2516772.

10. Srisaphonphusitti L, Manimmanakorn N, Manimmanakorn A, Hamlin MJ. Effects of Whole Body Vibration Exercise Combined with Weighted Vest in Older Adults: A Randomized Controlled Trial. *BMC geriatrics* (2022) 22(1):911.

11. Nithisup P, Manimmanakorn A, Hamlin MJ, Maneesai P, Manimmanakorn N, Khaengkhan C, et al. Exercise with Weight Vest Plus Chicken Protein Supplementation Delayed Muscle and Bone Loss in Older Female Adults. *Physical Activity and Nutrition* (2024) 28(4):15.

12. Greendale G, Hirsch S, Hahn T. The Effect of a Weighted Vest on Perceived Health Status and Bone Density in Older Persons. *Quality of Life Research* (1993) 2(2):141-52.

13. Hakestad KA, Torstveit M, Nordsletten L, Risberg M. Effect of Exercises with Weight Vests and a Patient Education Programme for Women with Osteopenia and a Healed Wrist Fracture: A Randomized, Controlled Trial of the Osteoactive Programme. *BMC musculoskeletal disorders* (2015) 16(1):352.

14. Bean JF, Kiely DK, LaRose S, O'Neill E, Goldstein R, Frontera WR. Increased Velocity Exercise Specific to Task Training Versus the National Institute on Aging's Strength Training Program: Changes in Limb Power and Mobility. *Journals of Gerontology Series A: Biomedical Sciences and Medical Sciences* (2009) 64(9):983-91.

15. Normandin E, Yow D, Crotts C, Kiel J, Beavers K, Nicklas BJ. Feasibility of Weighted Vest Use During a Dietary Weight Loss Intervention and Effects on Body Composition and Physical Function in Older Adults. *The Journal of frailty & aging* (2018) 7(3):198-203.
